# Effect of mucosal adjuvant IL-1β on heterotypic immunity in a pig influenza model

**DOI:** 10.3389/fimmu.2023.1181716

**Published:** 2023-04-20

**Authors:** Anna Schmidt, Basudev Paudyal, Sonia Villanueva-Hernández, Adam Mcnee, Eleni Vatzia, Brigid Veronica Carr, Selma Schmidt, Amy Mccarron, Veronica Martini, Silke Schroedel, Christian Thirion, Ryan Waters, Francisco J. Salguero, Wilhelm Gerner, Matthias Tenbusch, Elma Tchilian

**Affiliations:** ^1^ Virologisches Institut-Klinische und Molekulare Virologie, Universitätsklinikum Erlangen, Friedrich-Alexander-Universität (FAU) Erlangen-Nürnberg, Erlangen, Germany; ^2^ Host Responses, The Pirbright Institute, Pirbright, United Kingdom; ^3^ Institute for Research in Biomedicine, Bellinzona, Switzerland; ^4^ SIRION Biotech GmbH, Martinsried, Germany; ^5^ United Kingdom Health Security Agency, UKHSA-Porton Down, Salisbury, United Kingdom; ^6^ Medical Immunology Campus Erlangen (MICE), Friedrich-Alexander-Universität (FAU) Erlangen-Nürnberg, Erlangen, Germany

**Keywords:** influenza virus, pig, mucosal immunity, IL-1β, heterotypic protection, porcine B cells, porcine T-cells, lung-resident memory T-cells

## Abstract

T cell responses directed against highly conserved viral proteins contribute to the clearance of the influenza virus and confer broadly cross-reactive and protective immune responses against a range of influenza viruses in mice and ferrets. We examined the protective efficacy of mucosal delivery of adenoviral vectors expressing hemagglutinin (HA) and nucleoprotein (NP) from the H1N1 virus against heterologous H3N2 challenge in pigs. We also evaluated the effect of mucosal co-delivery of IL-1β, which significantly increased antibody and T cell responses in inbred Babraham pigs. Another group of outbred pigs was first exposed to pH1N1 as an alternative means of inducing heterosubtypic immunity and were subsequently challenged with H3N2. Although both prior infection and adenoviral vector immunization induced strong T-cell responses against the conserved NP protein, none of the treatment groups demonstrated increased protection against the heterologous H3N2 challenge. Ad-HA/NP+Ad-IL-1β immunization increased lung pathology, although viral load was unchanged. These data indicate that heterotypic immunity may be difficult to achieve in pigs and the immunological mechanisms may differ from those in small animal models. Caution should be applied in extrapolating from a single model to humans.

## 1 Introduction

Influenza virus infection remains a global health threat to humans, and animal influenza A virus is an important zoonotic pathogen with pandemic potential. However, current trivalent or quadrivalent inactivated influenza vaccines induce strain-specific neutralizing antibodies against the highly variable surface glycoprotein hemagglutinin (HA) and do not provide heterotypic protection against infection with influenza viruses from different HA subtypes. Furthermore, pandemics may cause devastating mortality so there is an urgent need to develop vaccines that provide broader protection and decrease the need for annual immunization.

Compared to inactivated vaccines, respiratory tract infection with a live influenza virus can induce cross-protective heterotypic immunity. The term “partial heterotypic immunity” (also called “heterosubtypic”) was introduced by Schulman and Kilbourne in 1965, who observed that mice infected with an H1N1 strain and subsequently challenged with a lethal H2N2 virus had reduced viral titers in the lung, milder lung pathology, and decreased mortality in the absence of neutralizing antibodies (1). Since then, the concept of heterotypic immunity has been confirmed in multiple animal models of influenza infections in the absence of cross-neutralizing antibodies (2). Investigation of the immunological mechanisms mediating cross-protection revealed the role of CD8 and CD4 T cells in recognizing conserved viral internal antigens such as nucleoprotein (NP) (3, 4). Heterotypic protection can be transferred in mice with internal protein-specific T cells (5). Correlative studies in humans also suggest that cross-reactive T cells provide partial protection against influenza infection (3, 6, 7).

More recently, it has been demonstrated that local lung-resident memory T cells (TRM) are essential for heterotypic immunity and the prevention of severe disease in respiratory infections in mice (8–11). TRM cells are most effectively induced by natural infection or respiratory delivery of vaccines. Although it was shown that local antigen expression together with local inflammation are essential for the induction of lung TRM, it is not clear what features of a mucosal vaccine are required for the optimal generation of TRM. It has been postulated that innate immune signaling through pattern recognition receptors is crucial for the efficient generation of adaptive immune responses (12). Inflammasome signaling during natural influenza infection results in, among other events, the local production of IL-1β and IL-18, which are essential for optimal adaptive immune responses to influenza virus infection (13, 14). Mucosal production of IL-1β attracts innate and adaptive immune cells by the induction of cytokines and adhesion molecules (15) but also has a direct proliferative effect on CD4 and CD8 T cells (16, 17). Intranasal delivery of IL-1β, together with HA and NP encoding adenoviral vectors (Ad-HA/NP+Ad-IL-1β), significantly increased the immunogenicity and heterotypic protection against the influenza virus in mice (11). The expression of IL-1β induced migration of dendritic cells and influenced TRM imprinting by generating tissue-related factors, like TGFβ, known to be a positive regulator of the adhesion molecule CD103.

Pigs, like humans, are natural hosts for the influenza A virus and display similar clinical manifestations and pathogenesis, making them an appropriate large animal model for studying influenza infection and new vaccine candidates (18, 19). Porcine and human lungs show many similarities and they share the same histological structure, epithelial lining, distribution of sialic acid receptors, and electrolyte transport (20). We have identified porcine TRM and B cells subsets and developed the tools to characterize their specificity, function, and distribution in the respiratory tract (21–23). In the present study, we evaluated the immunogenicity and protective efficacy of mucosal delivery of Ad-HA/NP+Ad-IL-1β to heterologous H3N2 challenge in pigs and compared this to heterotypic protection induced by a prior infection with pH1N1.

## 2 Materials and methods

### 2.1 Preparation of adenoviral vector vaccines

All adenoviral vector vaccines were replication-deficient (E1 and E3 deleted) and based on the human serotype Ad5. Ad-NP encoded a codon-optimized sequence of the nucleoprotein (NP) derived from H1N1/PR8/34 (NCBI: NP_040982.1) as described previously (11). Ad-HA encoded a codon-optimized sequence of the hemagglutinin (HA) derived from the pandemic strain pH1N1/Texas/05/2009 (GenBank: ACP41934.1). Ad-IL-1β encoded the mature form of porcine IL-1β (amino acids 115-267, NCBI: NP_999220.1) which was fused to a tPA signal peptide at the N-terminus for efficient secretion. The expression of the porcine IL-1β was verified by ELISA. An adenoviral vector lacking an insert was used as a control (Ad-empty). High-titer viral stocks were produced in collaboration with Sirion Biotech (Martinsried, Germany).

### 2.2 Influenza virus infection and immunization of pigs

Animal experiments were approved by the ethical review processes of the Pirbright Institute and the Animal and Plant Health Agency (APHA) according to the UK Animals (Scientific Procedures) Act 1986 under project license P47CE0FF2.

#### 2.2.1 Immunogenicity study with Babraham pigs

Eighteen 15 weeks old inbred Babraham pigs (bred at APHA Weybridge) with an average weight of 36.5 kg were screened for influenza A antibody-free status by HAI using H1N1pdm09, H1N2, H3N2, and avian-like H1N1. The animals were randomly assigned to four groups and treated as follows: 1) infected with 8x10^6^ PFU of MDCK grown A/swine/England/1353/2009 (pH1N1) - 2 ml per nostril using a mucosal atomization device (MAD, Wolfe-Tory Medical) (n=5); 2) immunized intranasally with 1x10^9^ particles of Ad-HA together with 1x10^9^ particles of Ad-NP referred to as (Ad-HA/NP) using MAD 2 ml per nostril (n=5); 3) immunized intranasally with a mix of 2x10^9^ particles of Ad-HA/NP and 1x10^9^ particles recombinant adenoviral vector encoding porcine IL-1β (Ad-HA/NP + Ad-IL-1β) (n=5) using MAD 2 ml per nostril or 4) controls given 2 ml per nostril of PBS using MAD (n=3). The animals were anesthetized prior to immunization with a mixture of 5 mg/kg Zoletil (2.5 mg/kg of Tiletamine + 2.5 mg/kg of Zolazepam) and 0.05 mg/kg Domitor (medetomidine). Following pH1N1 infection, daily nasal swabs were collected for seven days to assess the viral load. Blood samples were obtained on days 7, 14, and 21 post-infection/immunization. Three weeks after treatment all animals were humanely culled with an overdose of pentobarbital anesthetic and nasal turbinates (NT), bronchoalveolar lavage (BAL), tracheobronchial lymph nodes (TBLN), lung, spleen, and blood were collected.

#### 2.2.2 Efficacy study with outbred pigs

In total, 20 influenza free 7 weeks old Landrace × Large White pigs (outbred, average weight of 21 kg) were randomized into four groups of five and challenged with either pH1N1or immunized with Ad-HA/NP or Ad-HA/NP+Ad-IL-1β as described above for the Babraham pigs. Controls were immunized intranasally with 1x10^9^ empty adenoviral particles. Nasal swabs were collected daily for seven days from pigs infected with pH1N1. At 28 days post-infection/immunization, all pigs were inoculated intranasally with 2.6x10^7^ PFU of A/swine/Ohio/A01354299/2017(H3N2) - 2ml to each nostril using a MAD300 device. Clinical signs (temperature, loss of appetite, recumbence, skin hemorrhage, respiratory change, nasal discharge, and altered behavior) were observed and recorded daily. All the pigs were humanely killed 4 days after the H3N2 infection. This time point was chosen as it allows 4 days for monitoring virus shedding in daily nasal swabs, there is still significant viral load in the lungs and BAL and lung pathology is well developed (21, 24, 25). For logistical reasons, two H3N2 challenges were performed, with half of the animals challenged at 28 days and the remainder at 30 days after pH1N1 or vaccine exposure. As the analysis of samples from pigs challenged at days 28 and 30 did not show any significant differences, for simplicity in presentation, the results of the assays carried out on pigs challenged on both days have been amalgamated in all figures. At postmortem, blood, BAL, and lung were collected and processed as described before (24). Daily nasal swabs were collected after the H3N2 challenge for assessment of virus shedding by plaque assays. Viral load was also assessed in BAL and lung by plaque assays post H3N2 infection and qRT-PCR.

### 2.3 Virus titration and viral RNA detection

Virus titers in nasal swabs, BAL fluid, and lung accessory lobe were determined by plaque assay on MDCK cells as previously described (25). Viral RNA was extracted from BAL fluid and homogenized lung samples with QIAamp viral RNA mini kit (Qiagen) according to the manufacturer’s instructions. Samples were quantified by Reverse Transcription quantitative Polymerase Chain Reaction (RT-qPCR) (GoTaq 1-Step RT-qPCR kit, Promega) for the influenza A virus M gene using an MxPro 3500P instrument and MxPro analysis software (Agilent). Briefly, reverse transcription was performed at 37°C for 15 min followed by 10 min at 95°C; 2-step cycling was then performed with denaturation for 10 s at 95°C with annealing for 31 s at 60°C, extension at 72°C for 31 s and collection at 81°C for 31 s and this was repeated for 40 cycles. The results were expressed as the number of copies of RNA using a standard 10-fold dilution series of M gene standard RNA. For M vRNA analysis, the following primers were used: forward primer, 5’-AGA TGA GTC TTC TAA CCG AGG TCG-3’, two reverse primers, 5’-TGC AAA AAC ATC TTC AAG TCT CTG-3’ and 5’-TGC AAA GAC ATC TTC CAG TCT CTG-3’.

### 2.4 Serological assays

Determination of end point titer ELISAs and microneutralization (MN) assays on BAL and serum samples were performed using standard procedures (26–28). pH1N1 or H3N2 virus or recombinant hemagglutinin (HA) (1 µg/ml) protein from A/England/195/2009 (HA), H3 from A/Hong Kong/5738/14 (H3N2), H5 from A/ty/Turkey/1/05 (H5N1) provided by Alain Townsend (University of Oxford) or H7 from A/ty/Italy/984/00 (H7N1), provided by Florian Krammer (Mount Sinai), were coated overnight on 96-well microtiter plates (Nunc MaxiSorp, Sigma Aldrich).

### 2.5 Gross pathology and histopathological scoring of lung lesions

In the outbred heterologous infectious study, at *postmortem*, the lungs were removed, and digital photographs were taken of the dorsal and ventral aspects. Gross pathology was scored by quantitating the lesion areas as previously described by Halbur (29) and calculating the total area showing lesions by digital image analysis using Nikon NIS-Ar software (v 4.50). Lung tissue samples from the right cranial, middle, and caudal lung lobes were excised and fixed in 10% neutral buffered formalin for routine histological processing. Immunohistochemical detection of influenza A virus nucleoprotein (NP) was performed as previously described (24, 30). Stained tissue sections were scanned with a Hamamatsu Nanozoomer S360 digital scanner and e-slides were examined with ndp.view 2 software (v2.9.29). Histopathological changes in the H&E-stained lung tissue sections were scored by a veterinary pathologist blinded to the treatment group using five parameters (necrosis of the bronchiolar epithelium, airway inflammation, perivascular/bronchiolar cuffing, alveolar exudates, and septal inflammation) scored on a 5-point scale of 0 to 4 and then summed to give a total slide score ranging from 0 to 20 per slide and a total animal score from 0 to 60 (25). We have used the IHC component of the scoring system developed by Gauger et al. to evaluate NP expression in tissues (31).

### 2.6 Flow cytometry

Cryopreserved cells from NT, BAL, lung, TBLN, and PBMC were used for T cell phenotyping, tetramer staining, and intracellular staining for IFNγ, IL-2, and TNF as previously described (22, 24). Two million cells per well were seeded in R10 media (RPMI supplemented with 10% FCS, 1% Penicillin-Streptomycin, and 1% HEPES) in 96 well plates. Staining intensity was enhanced by incubation with 50 nM protein kinase inhibitor (PKI, Dasatinib BMS-354825, Sprycel) for 30 min at 37°C (32). Biotinylated monomers were assembled to multimers by successive addition of fluorochrome-coupled streptavidin-conjugates (Streptavidin-BV650, Streptavidin-BV421 (BioLegend). Tetramer staining was performed for NP_181-189 AAVGKVGTI_ (AAV) and NP_290-298 DFEREGYSL_ (DFE), 0.1 µg/µl for 30 min at 4°C. Surface staining to further phenotype the cells was carried out using anti-porcine CD3ϵ-APC, anti-pig CD8β-PE, anti-pig CD4a-PerCP-Cy5.5, anti-pig CD45RA-FITC, anti-human CCR7 -BV711 and LIVE/DEAD-APC-Cy7 (**Table 1**). The antibodies and the fixable near-IR LIVE/DEAD dye were added in staining buffer (PBS + 2% FCS + 1% 0.5M EDTA) and following incubation, cells were washed twice with PBS and fixed with 4% paraformaldehyde solution for 20 min at 4°C. Fixed cells were washed and resuspended in a staining buffer for measurement on BD LSRFortessa.

**Table 1:** Antibodies used:

Antigen	Clone	Isotype	Fluorochrome	Source of primary Ab	Details of secondary labelling	
Tetramer staining						
CD3ϵ	PPT3	Mouse IgG1	APC	Southern Biotech		
CD8β	PPT23	Mouse IgG1	PE	Bio-Rad		
CD4	74-12-4	Mouse IgG2b	PerCP-Cy5.5	BD Biosciences		
CD45RA	MIL13	Mouse IgG1	FITC	Bio-Rad		
CCR7 (CD197)	3D12	Rat IgG2a	BV711	BD Biosciences		
Intracellular cytokine staining						
CD8β	PPT23	Mouse IgG1	FITC	Bio-Rad		
CD4	74-12-4	Mouse IgG2b	PerCP-Cy5.5	Bio-Rad		
IFNγ	P2G10	Mouse IgG1	AF647	BD Biosciences		
TNFα	Mab11	Mouse IgG1	BV421	BioLegend		
IL-2	A150D3F1	Mouse IgG2a	PE-Cy7	Invitrogen	Anti-mouse IgG2a, clone RMG2a-62, BioLegend	
B cell staining with HA-probe						
CD79α	HM47	Mouse IgG1	PerCP-Cy5.5	BioLegend		
Blimp1	3H2E8	Mouse IgG1	AF647	Santa Cruz Biotechnology		
IRF4	3E4	Rat IgG1	PE	ThermoFisher		
Bcl-6	K112-91	Mouse IgG1	BV421	BD Biosciences		
Pax5	1H9	Rat IgG2a	BV510	BD Biosciences		
Tfh staining						
CD3ϵ	BB23-8E6-8C8	Mouse IgG2a	PerCP-Cy5.5	BD Biosciences	
CD4	74-12-4	Mouse IgG2b	AF488	Southern Biotech	Goat anti-mouse IgG2b, Jackson
ICOS (CD278)	C398.4A	Hamster IgG	BV605	BioLegend	
Bcl-6	K112-91	Mouse IgG1	BV421	BD Biosciences	

Influenza-specific T cell responses were evaluated by analysis of cytokine production of cells isolated from BAL, lung, TBLN, and PBMC. One million cells per well were seeded in 96 well plates and stimulated with H1N1pdm09 (MOI = 1) overnight at 37°C. Positive controls were stimulated with 12-myristate 13-acetate (PMA) and ionomycin Cell Stimulation Cocktail (Invitrogen). Four hours prior to the cell staining, all cells were treated with protein transport inhibitor BD GolgiPlug containing brefeldin A (BD Cytofix/Cytoperm Plus Kit, BD Biosciences). After stimulation, cells were washed and stained with anti-pig CD8β-FITC, anti-pig CD4a-PerCP-Cy5.5, and LIVE/DEAD-APC-Cy7. Intracellular cytokine staining was performed using Fixation/Permeabilization Kit (BD Cytofix/Cytoperm Plus, BD Biosciences) following the manufactures instructions. Anti-pig IFNγ-AF647, anti-human TNFα-BV421, and anti-pig IL-2 were added. Anti-mouse IgG2a-PE/Cy7 was used for the detection of IL-2. Cells were washed and data were acquired on a MACSQuant Flow Cytometer (Miltenyi Biotec) and analyzed using FlowJo software (Tree Star Inc.).

For the B cell staining, freshly isolated cells were initially labeled with fixable near-IR LIVE/DEAD dye (ThermoFisher) in PBS, followed by fixation and permeabilization with the eBioscience™ Foxp3 staining buffer set (ThermoFisher). Thereafter, total B cells and B cell subsets were labeled with the antibodies listed in **Table 1**. In parallel, a biotinylated HA-probe was added and, in a final incubation step, visualized using streptavidin-PE-Cy7 (ThermoFisher). After two final washes, the cells were analyzed on a MACSQuant10 flow cytometer (Miltenyi Biotec). The staining of cells for the identification of Tfh cells followed the same protocol, but antibodies against CD3, CD4, CD8α, and ICOS (**Table 1**) were added in the first incubation step, followed by a second incubation with fixable near-IR LIVE/DEAD dye and streptavidin-PE-Cy7 (same reagents as above). Following fixation and permeabilization, cells were incubated with antibodies against Bcl-6. Cells for Tfh cell analysis were recorded on a BD LSR Fortessa (BD Biosciences). Each incubation step prior to fixation lasted 20 min and the fixation and intracellular staining steps lasted 30 min. For both samples, at least 2x10^5^ cells were acquired on the respective flow cytometer.

### 2.7 Statistical analysis

Statistical analyses were performed using GraphPad Prism 8.1.2 (GraphPad Software, Inc). The results are shown as mean ± SEM. The data were first analyzed for normality. Normally distributed data sets were subjected to a one-way ANOVA/two-way ANOVA with Tukey’s multiple comparisons test, while non-normally distributed data sets were subjected to a Kruskal-Wallis test with Dunn’s multiple comparisons test. A *p-*value of <0.05 was considered to be statistically significant.

## 3 Results

### 3.1 Experimental design and antibody responses following influenza infection or immunization with IL-1β

**Figure 1:**
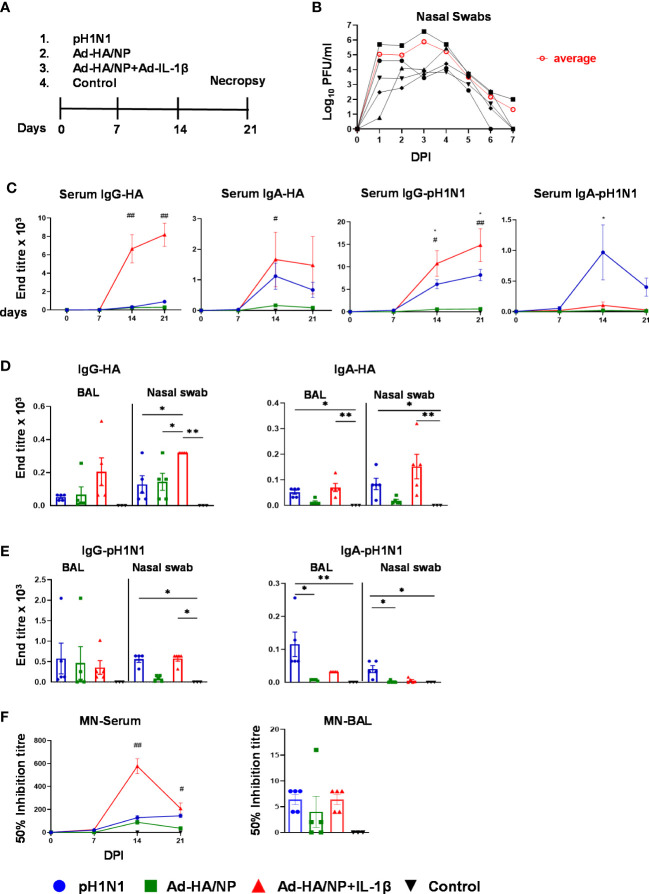
Experimental design, viral load, and systemic as well as mucosal antibody responses. **(A)** Babraham pigs (five per group) were either intranasally infected with the pH1N1 virus or immunized with Ad-HA/NP or Ad-HA/NP+Ad-IL-1β. Control animals were given PBS intranasally (three pigs). Nasal swabs were collected daily for seven days. Weekly blood samples were collected. All animals were humanely culled on day 21 post-infection or immunization. **(B)** Virus load in nasal swabs, taken daily after challenge, were assessed by plaque assay. Black lines represent the viral load in individual pigs and the red line indicates the mean viral load of the five animals in the pH1N1 group. **(C)** HA- and pH1N1-specific IgG and IgA responses in serum were determined by ELISA at the indicated time points. The mean ± SEM of five or three (control) pigs per group are shown at the indicated points. **(D, E)** HA- and pH1N1-specific IgG and IgA responses in BAL and nasal swabs were analyzed by ELISA at day 21 post-infection or immunization only. Each symbol represents an individual animal, the top of the bar the mean, and the line the standard error (SEM). **(F)** Functional antibody responses against pH1N1 were assessed in BAL at day 21 and in serum over time by microneutralization assay. For serum, the mean ± SEM of five or three (control) pigs per group are shown. For BAL each symbol represents an individual animal, the top of the bar the mean, and the line the standard error (SEM). (MN). **(C–F)** Statistical significances were analyzed for serum by two-way ANOVA followed by Tukey’s Multiple Comparison Test. One-way ANOVA was used for IgG-HA (BAL and nasal swabs) and Kruskal–Wallis test with Dunn’s multiple comparisons test was used for IgA-HA, IgG-pH1N1, IgA-pH1N1 (BAL and nasal swabs). Asterisks or hashtags denote significance between indicated groups (*, p<0.05 and **, p<0.01) and #, p<0.05 Ad-HA/NP + Ad-IL-1β vs. Ad-HA/NP, control; ##, p<0.05 Ad-HA/NP + Ad-IL-1β vs. all groups.

To assess the effect of mucosal delivery of IL-1β on immune responses, inbred Babraham pigs (33, 34) were immunized intranasally with recombinant adenoviral vectors expressing NP from H1N1/PR8/34 virus and HA from pH1N1/Texas/05/2009 (Ad-HA/NP) in the presence or absence of Ad-IL-1β and compared to, animals infected intranasally with A/swine/England/1353/2009 (pH1N1). Control pigs were given PBS intranasally (**Figure 1A**). The animals were culled three weeks later, and the immune responses in nasal turbinates (NT), bronchoalveolar lavage (BAL), lung, tracheobronchial lymph nodes (TBLN) and blood were evaluated. Virus shedding from pH1N1-infected pigs was detected in nasal swabs taken daily for the first 7 days in agreement with previous studies (35) (**Figure 1B**).

Antibody responses in serum, BAL, and nasal swabs were evaluated by ELISA against recombinant HA from A/England/195/2009 (HA) or pH1N1 virus (**Figures 1C–E**). Intranasal delivery of Ad-HA/NP+Ad-IL-1β significantly increased HA-specific IgG and IgA serum responses compared to Ad-HA/NP-only immunized or pH1N1-infected animals (**Figure 1C**). Serum pH1N1-specific IgG levels were also increased in the Ad-HA/NP+Ad-IL-1β group, while pH1N1 infection increased the pH1N1-specific IgA titers.

IgG and IgA levels were measured in BAL and nasal swabs at the time of culling 21 days post-immunization or infection (**Figure 1D**). Significantly higher HA-specific IgG responses were measured in nasal swabs in animals immunized with Ad-HA/NP+Ad-IL-1β compared to Ad-HA/NP only or pH1N1 infection and had higher IgA responses compared to the controls. Ad-HA/NP+Ad-IL-1β administration also significantly increased pH1N1-specific IgG titers in nasal swabs (1:576) reaching the titers measured after pH1N1 infection (1:560), which were significantly higher compared to titers in controls (**Figure 1E**). The pH1N1-specific IgA titers were significantly increased in serum, BAL, and nasal swabs of pH1N1-infected animals compared to Ad-HA/NP and controls (**Figures 1C, E**). Serum 50% neutralizing inhibition titers were significantly greater in the Ad-HA/NP+Ad-IL-1β group (1:704) compared to pH1N1-infected (1:256) and Ad-HA/NP-immunized animals (1:200) 14 days post-infection (**Figure 1F**), while no significant differences in neutralizing activity were detected in BAL.

In summary, these results indicate that mucosal delivery of Ad-HA/NP+Ad-IL-1β increased HA-specific IgG and IgA titers in serum and nasal swabs, and IgG responses in BAL, while pH1N1 infection resulted in higher IgA pH1N1 responses in serum, BAL, and nasal swabs.

### 3.2 B and Tfh cell responses after immunization or infection of Babraham pigs

Next, we quantified B cell responses as recently described (23, 36). Total B cells were identified by co-expression of CD79α and Pax5 and plasma cells and plasmablasts by the co-expression of Blimp-1 and IRF4 (**Supplementary Figure 1A**). B cells that did not express these transcription factors were analyzed for Bcl-6, thought to represent porcine germinal center (GC) B cells (36, 37). The remaining B cells (CD79α^+^Pax5^+^Blimp-1^-^IRF4^-^Bcl-6^-^) were a mixture of naïve, memory, and recently activated B cells. Total B cells were more frequent in TBLN and NT compared to blood or BAL but no significant differences between treatment groups were detected (**Supplementary Figure 1B**). CD79α^+^Pax5^+^Blimp-1^+^IRF4^+^ plasma cells/plasmablasts were enriched in BAL and NT relative to blood and TBLN but also this phenotype did not differ between groups. GC B cells with a CD79α^+^Pax5^+^Blimp-1^-^IRF4^-^Bcl-6^+^ were most frequent in TBLN and were significantly higher in the pH1N1-infected pigs compared to the controls (**Supplementary Figure 1B**).

**Figure 2:**
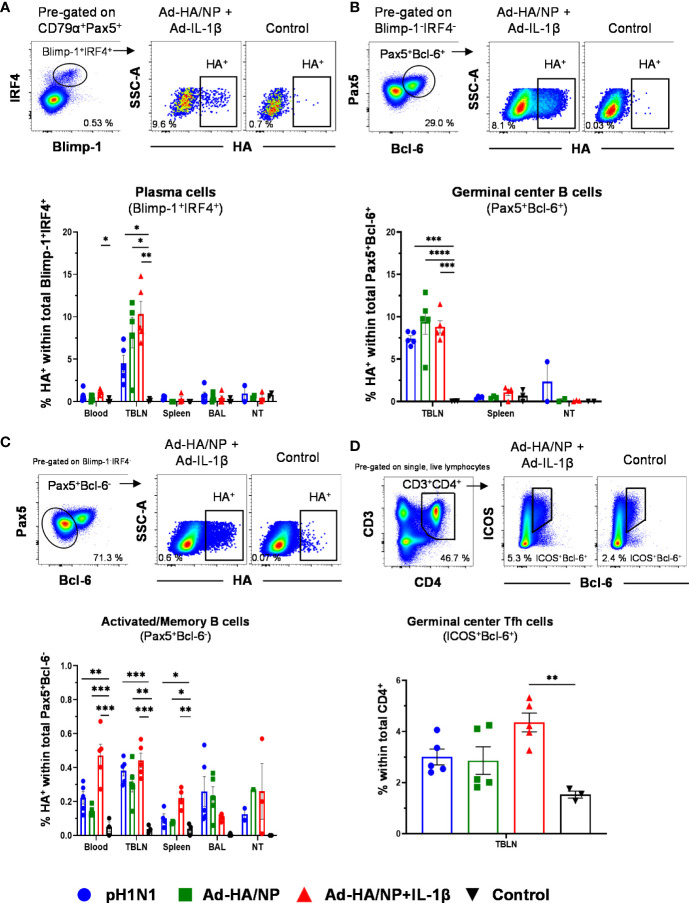
Frequencies of HA-specific plasma cells, germinal center B cells, and activated/memory B cells. B cell subsets were gated as described in **Supplementary Figure 1**. **(A)** Plasma cells, **(B)** GC B cells, and **(C)** activated/memory B cells and HA-binding cells were identified within these subsets. Representative raw data is shown for an Ad-HA/NP + Ad-IL-1β treated pig versus a control pig. Graphs below show percentages of HA-binding cells within the respective subset across tissues and treatment groups. **(D)** CD3^+^CD4^+^ICOS^+^Bcl-6^+^ Tfh cells were identified according to the gating on the top. Each symbol represents data from an individual pig of the different treatment groups. Asterisks indicate significant differences between treatment groups within one location (*, *p*<0.05; **, *p*<0.01; ***, *p*<0.001 and ****p<0.0001).

Next, we investigated HA-specific B cells using a biotinylated HA probe. Within plasma cells/plasmablasts in TBLN, the proportion of HA-binding cells was significantly higher in the two Ad-immunized groups, compared to the control pigs. In addition, Ad-HA/NP+Ad-IL-1β-immunized pigs had significantly more HA-specific plasma cells than pH1N1-infected pigs (**Figure 2A**). In the blood, Ad-HA/NP+Ad-IL-1β immunization induced significantly more plasma cells/plasmablasts than control (**Figure 2A**). HA-binding GC B cells in TBLN were significantly higher in all three treatment groups compared to the controls (**Figure 2B**). In regard to recently activated or memory B-cells (CD79α^+^Pax5^+^Blimp-1^-^IRF4^-^Bcl-6^-^ phenotype), the results were more heterogenous (**Figure 2C**). This subset of HA-specific B cells was significantly enriched in blood and spleen of Ad-HA/NP+Ad-IL-1β-immunized pigs compared to all other animals. Additionally, the infected or immunized animals had higher frequencies of HA-binding cells in TBLN than the control pigs. Similar results were found in the BAL, although significance was not reached.

Having identified a substantial proportion of HA-binding cells among GC B cells in TBLN we focused on these lymph nodes and investigated total T follicular helper (Tfh) cells identified by a CD3^+^CD4^+^ICOS^+^Bcl-6^+^ phenotype (36) (**Figure 2D**). Similar to HA-binding GC B cells, significantly enriched frequencies of Tfh cells were detected in the Ad-HA/NP+Ad-IL-1β-immunized animals in comparison to the control pigs (**Figure 2D**).

In summary, these results show that different B cell subsets and TBLN-residing Tfh cells responded to immunization or pH1N1 infection and that the higher frequencies HA-binding plasma cells in the TBLNs and blood of the Ad-HA/NP+Ad-IL-1β-immunized pigs correlated with high HA IgG titers in serum of this treatment group.

### 3.3 T cell responses following infection or immunization

**Figure 3:**
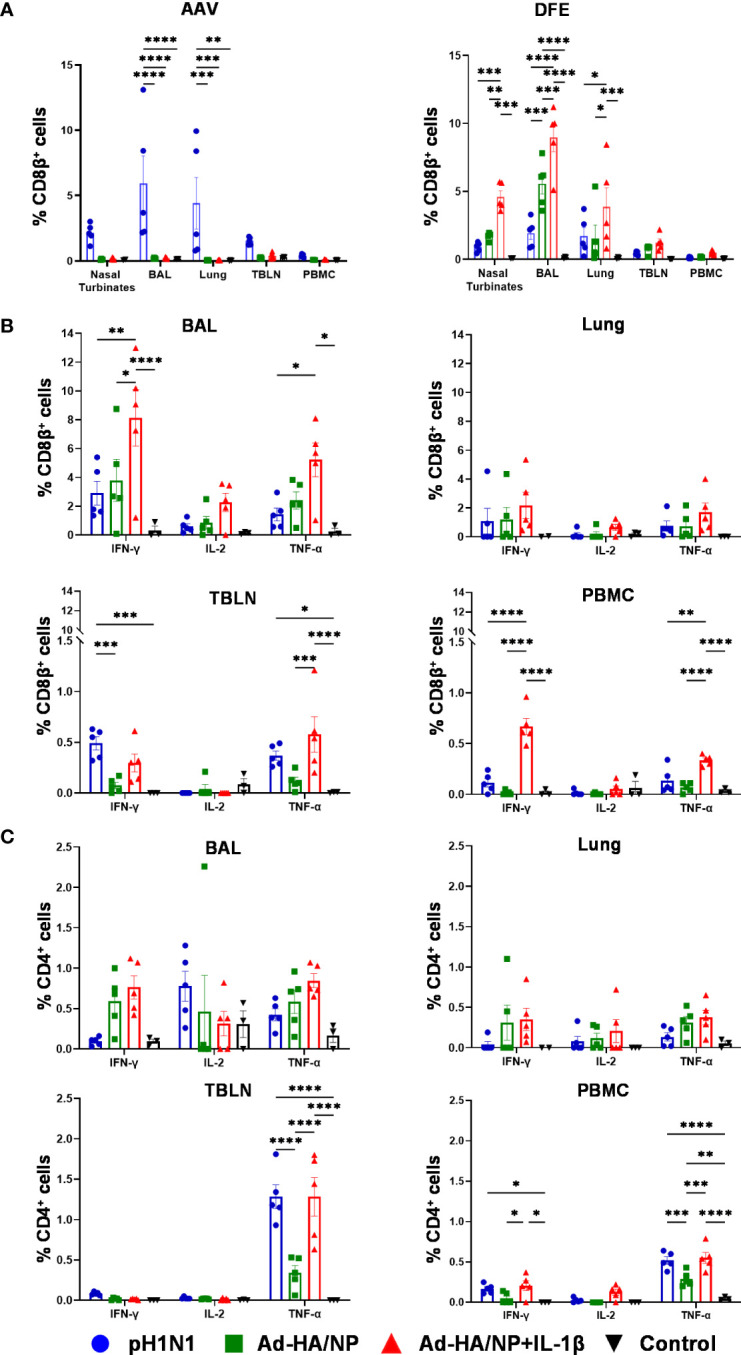
Tetramer recognition and functional profile of porcine T cells in different tissues. **(A)** Proportions of AAV- (left) and DFE- (right) specific CD8β^+^ T cells in different tissues of pH1N1 infected or Ad-HA/NP ± Ad-IL-1β immunized pigs at day 21. IFNγ, IL-2, and TNFα cytokine secretion were measured by intracellular staining in **(B)** CD8^+^ and **(C)** CD4^+^ T cells from the indicated tissues at day 21 post-immunization or infection. Statistical significances were analyzed by two-way ANOVA followed by Tukey’s Multiple Comparison Test. Asterisks denote significance between indicated groups (*, *p*<0.05; **, *p*<0.01; ***, *p*<0.001 and ****p<0.0001).

The Babraham inbred pigs allowed us to enumerate influenza NP-specific CD8^+^ T cell responses in NT, lung, BAL, TBLN, and PBMC using two NP epitope tetramers: NP_181-189_ AAVGKVGTI (AAV) and NP_290-298_ DFEREGYSL (DFE) as previously described (22) (**Figure 3A**). AAV-recognition was detected only in the pH1N1-infected animals, but not in the two Ad-HA/NP immunized groups most likely because of the one amino acid difference between the NP from pH1N1 and NP from H1N1/PR8/34 which was used in the Ad-HA/NP vaccine (position 189 I in pH1N1 and 189 M in the Ad vaccine). The AAV responses were greatest in BAL (5.9%) and lung (4.4%), followed by nasal turbinates, TBLN, and PBMC.

DFE is conserved between the NP in pH1N1 and PR8 and a DFE-specific response was detected in all immunized or infected animals (**Figure 3A**). The highest frequencies of DFE-specific cells were detected in the BAL of Ad-HA/NP+Ad-IL-1β group: 9% (Ad-HA/NP+Ad-IL-1β), 5.6% (Ad-HA/NP), 2% (pH1N1), followed by nasal turbinates and lung. The frequencies of DFE-specific CD8β^+^ T cells in PBMC were 0.5% (Ad-HA/NP+Ad-IL-1β), 0.15% (Ad-HA/NP), 0.13% (pH1N1), and in TBLN were 1.24% (Ad-HA/NP+Ad-IL-1β), 0.75% (Ad-HA/NP), 0.46% (pH1N1). We further analyzed the expression of CD45RA and CCR7 on DFE CD8β^+^ T cells which identify naïve CD8β^+^ T cells (naïve, CD45RA^+^ CCR7^+^), central memory T cells (TCM, CD45RA^-^ CCR7^+^), effector memory T cells (TEM, CD45RA^-^ CCR7^-^) and differentiated effector T cells (TDE, CD45RA^+^ CCR7^-^) (**Supplementary Figure 2A**) (22). Ad-HA/NP+Ad-IL-1β immunization elicited the highest proportion of TEM in TBLN compared to the other groups, while TEM in PBMC were the highest in pH1N1 and Ad-HA/NP+Ad-IL-1β compared to the Ad-HA/NP only immunized group (**Supplementary Figure 2B**).

T cell responses were also analyzed by intracellular cytokine staining (ICS) in BAL, lung, TBLN, and PBMC (**Figures 3B, C**). IFNγ, TNF, and IL-2 production by CD4^+^ and CD8β^+^ T cells were measured following *ex vivo* pH1N1 stimulation. Ad-HA/NP+Ad-IL-1β immunization induced significantly higher frequencies of IFNγ and TNF producing CD8β^+^ T-cells in BAL and PBMC compared to the other groups. Pandemic-H1N1 infection and Ad-HA/NP+Ad-IL-1β induced comparable CD8 responses in TBLN. No differences in cytokine-producing CD4^+^ T cells were detected between the groups in BAL and lung (**Figure 3C**). However, TNF production in CD4^+^ T cells was the highest in the TBLN and PBMC of the pH1N1 and Ad-HA/NP+Ad-IL-1β groups, compared to the Ad-HA/NP group. There was also greater production of IFNγ in PBMC of Ad-HA/NP+Ad-IL-1β compared to Ad-HA/NP.

Overall, these results demonstrate that intranasal administration of Ad-HA/NP+Ad-IL-1β induced the highest proportion of DFE-specific NP cells in the respiratory tract as well as IFNγ and TNF producing CD8β^+^ T cells in BAL and PBMC. In contrast, pH1N1 infection induced the greatest AAV NP response and strong CD8 TNF production in TBLN and CD4 TNF in TBLN and PBMC.

### 3.4 Protective efficacy against heterologous H3N2 influenza virus challenge

**Figure 4:**
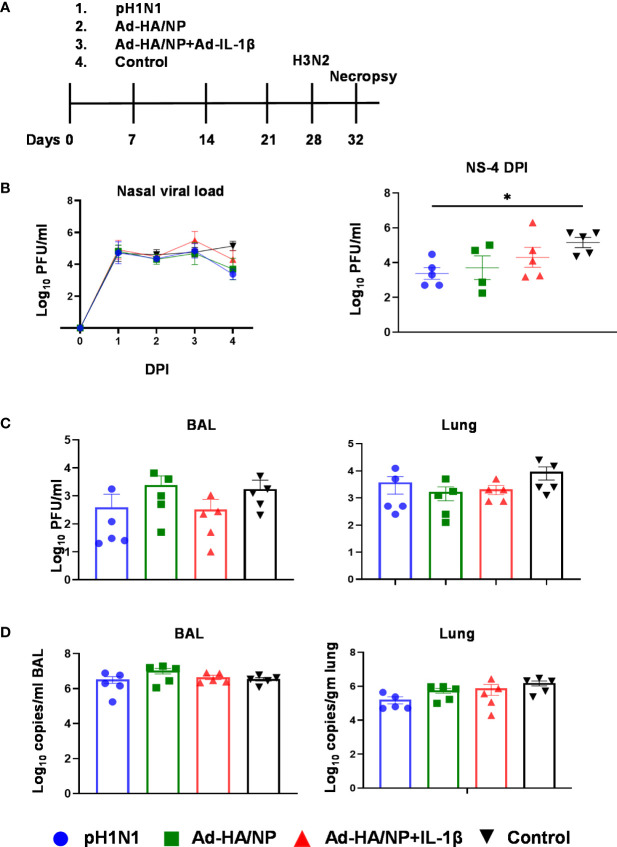
Experimental design of heterologous challenge study and viral load in nasal swabs, BAL, and lung. **(A)** Outbred pigs were intranasally infected with the pH1N1 or immunized with Ad-HA/NP or Ad-HA/NP + Ad-IL-1β or an empty vector as control. At 28 days post-infection or immunization, all pigs were challenged with the H3N2 virus and were culled 4 days later. Following H3N2 infection, nasal swabs (NS) were taken at 0, 1, 2, 3, and 4 DPI. **(B)** Virus load in daily nasal swabs and at day 4 post-infection **(C)** Virus load in BAL and lung assessed by plaque assay or **(D)** by RT-PCR. All values represent the mean ± SEM of five pigs per group (n=5). Statistical significances were analyzed by two-way ANOVA followed by Tukey’s Multiple Comparison Test on viral load over time **(B)** and Kruskal-Wallis test with Dunn’s multiple comparison test on **(C, D)**. Asterisks denote significant differences *, p<0.05 pH1N1 vs. control.

After analyzing the immunogenicity of Ad-HA/NP and Ad-HA/NP+Ad-IL-1β in inbred Babraham pigs we assessed their efficacy against heterologous H3N2 influenza virus challenge in outbred pigs. Outbred pigs were inoculated intranasally with either pH1N1 or immunized intranasally with Ad-HA/NP or Ad-HA/NP+Ad-IL-1β as described above. Control animals were immunized with an empty adenoviral vector. The pigs infected with pH1N1 shed the virus in the first 7 days as in the previous study using inbred Babraham pigs (data not shown). All the pigs were inoculated with A/swine/Ohio/A01354299/2017 (H3N2) 28 days post-infection or immunization. Rectal temperatures were elevated up to 40°C in all groups compared to the basal level of 38.5°C before the H3N2 challenge (data not shown). A single pig from the Ad-HA/NP+Ad-IL-1β group showed inappetence and cough between 1 to 3 days post-infection (DPI). Pigs were culled 4 days after the H3N2 challenge for evaluation of viral load, pathology, and immune responses (**Figure 4A**).

All animals shed the virus in daily nasal swabs but shedding was reduced in the group infected with pH1N1 compared to the control Ad group at four days post H3N2 challenge (p=0.016) (**Figure 4B**). Viral load in BAL and lung was similar between the groups as determined by plaque assays and qRT-PCR (**Figures 4C, D**).

**Figure 5:**
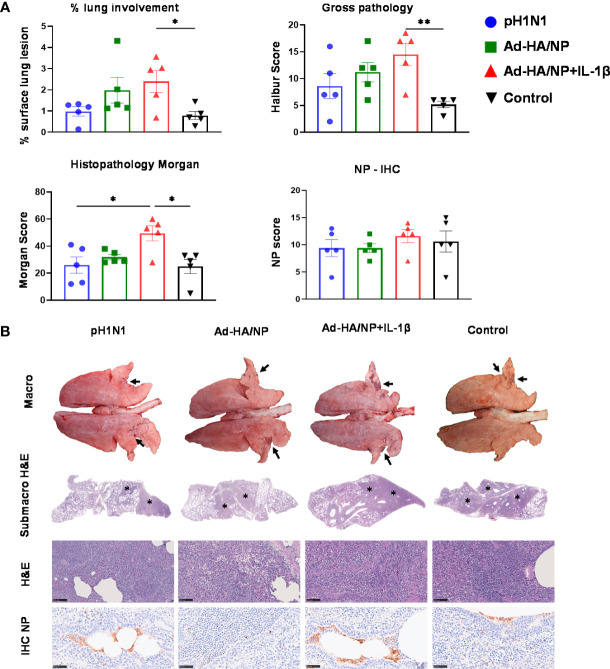
Pathological assessment of lung sections after heterologous challenge. Pigs were challenged with H3N2 28 days post pH1N1 infection or immunization with Ad-HA/NP or Ad-HA/NP+IL-1β or control. Four days after the H3N2 challenge, the animals were culled and lungs were scored for the appearance of gross and histopathological lesions. **(A)** The gross and histopathological scores for each individual in a group and the group means are shown, including the percentage of lung surface with lesions, the lesion scores, and histopathological Morgan scores. **(B)** Representative lung gross pathology, histopathology (submacroscopic and microscopic), and immunohistochemical NP staining of each group. Gross pathology is observed as areas of consolidation (arrows). At submicroscopic histopathology, areas of bronchopneumonia are characterized by the obliteration of the normal airway structures, with inflammatory cell infiltration (*), that can be observed at the microscopic level as bronchiolo- and broncho-interstitial pneumonia with necrosis of epithelial cells and inflammatory infiltrates in the airways and parenchyma (*). Virus NP detected by IHC (brown stain, arrows) within the bronchiolar wall and luminae and occasionally within the parenchyma. Bar = 100 micrometers. Pathology scores were analyzed using one-way ANOVA followed by Tukey’s multiple comparison test. Asterisks denote significant differences **, p<0.01 and *, p*<*0.05 versus the group indicated by the horizontal line.

Following the H3N2 challenge, all the animals developed typical gross lung lesions indicative of influenza infection as previously reported (38) (**Figure 5**). The Ad-HA/NP+Ad-IL-1β immunized group showed the highest gross pathology and histopathology scores (**Figure 5A**). Representative lung gross pathology, histopathology, and immunohistochemical NP staining are shown (**Figure 5B**). The macroscopic lesions were in areas of tissue consolidation, appearing purple and firm and generally well demarcated from the non-affected areas. Gross pathology was mostly observed in cranial and medial lobes, with less involvement of the caudal or accessory lobes. There was a significantly higher lung gross lesion area in the Ad-HA/NP+Ad-IL-1β group compared to the control group (p=0.041). Similarly, Ad-HA/NP+Ad-IL-1β induced the highest gross pathology as indicated by the Halbur scores (p=0.012). At the microscopic level, the lesions were typical of a viral bronchiolar and broncho-interstitial pneumonia characterized by the attenuation and necrosis of the respiratory epithelium. The obliteration of the normal respiratory tree with inflammatory cell infiltration within the parenchyma and airways was observed in all groups, while the Ad-HA/NP+Ad-IL-1β group showed increased histopathological lesions and histopathology scores compared to the control (p=0.015) or pH1N1-infected animals (p=0.02) (**Figures 5A, B**). There was no difference in NP expression measured by IHC between groups (**Figure 5A**).

Overall, these results indicate that neither prior pH1N1 exposure nor immunization with Ad-HA/NP increased protection against heterologous H3N2 influenza infection, although viral shedding was reduced at day four in the pH1N1 group. Ad-HA/NP+Ad-IL-1β immunization enhanced lung pathology although no increase in viral load in lungs or BAL was detected.

### 3.5 Immune responses after the H3N2 challenge

**Figure 6:**
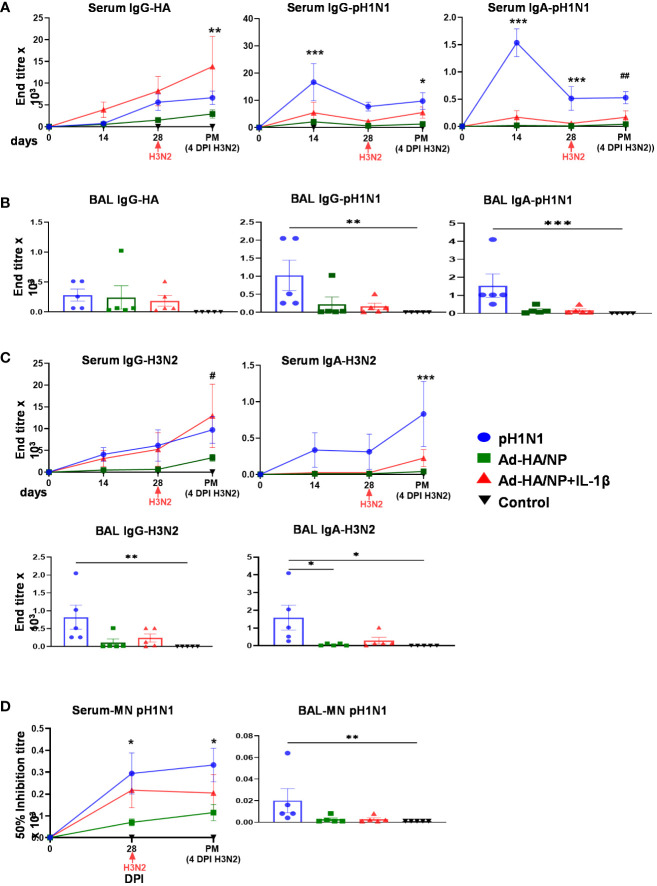
Antibody responses against pH1N1 and H3N2. IgG and IgA responses against the HA protein and pH1N1 were assessed in **(A)** serum at the indicated time points and in **(B)** post mortem BAL samples. **(C)** IgG and IgA responses against H3N2 were assessed in serum samples at day 14, 28 and 32 and in BAL samples post mortem. **(D)** Neutralizing antibody responses against pH1N1 were analyzed in BAL at day 32 and in serum at day 0, 28 and 32. All values represent mean ± SEM of five pigs per group (n=5). Statistical significances were analyzed by two-way ANOVA followed by Tukey’s Multiple Comparison Test for serum samples or Kruskal-Wallis test with Dunn’s multiple comparison test for BAL samples. ***, p<0.001 H1N1pdm09 vs. all groups; *, p<0.05 H1N1pdm09 vs. Ad-HA/NP; **, p<0.01 Ad-HA/NP vs. Ad-HA/NP + Ad-IL-1β ; #, p<0.05 Ad-HA/NP + Ad-IL-1β vs. Ad-HA/NP and control; ##, p<0.05 pH1N1 vs. Ad-HA/NP and control.

Circulating antibody responses were analyzed in serum over the time course of the study and in BAL at the time of culling at four days post H3N2 infection. After primary pH1N1 infection or immunizations, there was a gradual increase in HA-specific IgG in serum and although this was highest in the Ad-HA/NP+Ad-IL-1β group, this did not reach statistical significance in contrast to what we observed before. Serum HA-specific responses were, however, significantly higher in the Ad-HA/NP+Ad-IL-1β group at 4 days post H3N2 infection compared to the Ad-HA/NP group (**Figure 6A**). Pandemic-H1N1 infection induced the highest pH1N1-specific IgG and IgA responses in both serum and BAL (**Figures 6A, B**). Serum H3N2-specific IgG responses were significantly increased in the pH1N1 and Ad-HA/NP+Ad-IL-1β groups compared to the Ad-HA/NP and control groups at 4 DPI with H3N2 (**Figure 6C**). H3N2-specific IgA was significantly higher only in the pH1N1-infected animals, as were the H3N2-specific IgG and IgA responses in the BAL (**Figure 6C**).

After the initial infection/immunization, there was an increase in neutralizing titers to pH1N1, which was significantly higher in the pH1N1-infected group at day 28 and 4 days post H3N2 compared to the immunized and control groups (p<0.01) (**Figure 6D**). Neutralizing pH1N1 activity was also detected in BAL of pH1N1 infected pigs but at a very low level. No neutralizing activity against H3N2 was detected in BAL of the Ad-HA/NP and Ad-HA/NP+Ad-IL-1β immunized groups. There was serum IgG binding to H5 from A/ty/Turkey/1/05 (H5N1), but not to the H3 from A/Hong Kong/5738/14 (H3N2) or H7 from A/ty/Italy/984/00 (H7N1) suggesting cross-reactivity to other HAs from group 1 influenza viruses, but not from group 2 (**Supplementary Figure 3**).

## 4 Discussion

A broadly protective influenza A vaccine requires the induction of immunity to conserved components of the virus that either prevent infection or limit viral replication. Here we examined the protective efficacy of mucosal delivery of adenoviral vectors expressing HA from pH1N1/Texas/05/2009 and NP from H1N1 PR8 virus against heterologous H3N2 challenge in pigs. Mucosal delivery of Ad-NP/HA was protective in mice against homologous and heterologous H1N1, pH1N1, H3N2, and H7N7 strains (11). Protection was significantly improved by mucosal co-delivery of Ad-IL-1β which correlated with superior antibody and T cell responses. Inhibition of egress of circulating T cells from the lymph nodes with FTY720 during the heterologous infection had no impact on the degree of protection suggesting that protection was mediated by the local TRM (11). Therefore, we evaluated the effect of mucosal co-delivery of porcine IL-1β on local and systemic B and T cell responses in inbred Babraham pigs and compared it to immunity induced by natural pH1N1 infection.

Here we show that as in mice, intranasal delivery of Ad-HA/NP+Ad-IL-1β increased NP-specific CD8^+^ T cell frequencies, T cell cytokine production, and antibody responses. Nearly 10% of BAL CD8^+^ T cells were specific for NP as indicated by tetramer staining, which was comparable to pigs with a pH1N1 infection. Since the epitopes of NP recognized by vaccine- or infection-induced T cells differed slightly, intracellular cytokine staining after restimulation with whole virus particles allowed a better comparison of the total NP-specific T cell response. Again, the co-delivery of IL-1β significantly improved the local and systemic CD8^+^ T cell response after adenoviral vector immunization. Interestingly, the frequencies of IFNγ and TNF-producing NP-specific T cells in the BAL were superior to the ones observed after natural infection, confirming the potent adjuvant effect of IL-1β on local TRM formation in pigs. Analysis of accompanying B and Tfh cell responses indicates a considerable increase in HA-specific plasma, plasmablast, and GC B cells together with Tfh cells following intranasal infection or immunization. This is reminiscent of findings in the mouse model of influenza, where after intranasal infection an active GC reaction was found for up to five months in mediastinal lymph nodes, with the peak of the GC response occurring 3 to 4 weeks post-infection (39). Overall, it became clear that IL-1β potentiated immune responses.

However, the increased antibody, T cell, and B cell responses in Babraham pigs did not translate into better protection against heterologous H3N2 challenge in outbred pigs. Neither intranasal immunization with Ad-HA/NP+Ad-IL-1β nor prior pH1N1 exposure protected pigs against H3N2. In contrast to a previous study (35), antibody responses to the pH1N1 infection differed in Babraham and outbred pigs, which might be related to differences in age. In the present study, Babraham pigs were 15 weeks whereas the outbred pigs were 7 weeks of age. Although the antibody responses differed slightly between the two pig models, the co-delivery of IL-1β substantially increased the serum HA IgG titers in both models. Importantly, no neutralizing antibodies to the challenge virus H3N2 could be detected. Also, the strong local vaccine-induced TRM responses did not efficiently limit viral replication in the airways and had no beneficial impact on the disease progression or pathology in the absence of neutralizing antibodies, which is in sharp contrast to the results seen in the mouse model (8–11). However different assays and time points were employed in the mouse and pig influenza infectious studies: daily weight loss and viral load in BAL at days 3 and 7 post-infection were measured in mice. Daily nasal shedding up to day 4 post-infection, the viral load in BAL and lung, and lung pathology at day 4 post-infection were assessed in pigs. Although these assays and timing are different the evidence indicates that mice are better protected by immunization in the presence of IL-1β, while the pig data does not support this conclusion. Furthermore, in the future, it will be important to determine whether a single immunization with Ad-NP/HA will protect against the homologous challenge to pH1N1 and whether altering the extent of homology in the HA could induce cross-protection within the virus subtype.

Importantly prior pH1N1 infection did not reduce lung pathology following the H3N2 challenge, although viral shedding was reduced at 4 days post-H3N2, but not the viral load in the lung or BAL. Similar results were reported by Qiu et al., demonstrating that cross-protection against H3N2 was minimal in pH1N1 or H1N1 immune pigs (40). However, cross-protection was almost complete in H1N2-pre-exposed pigs, suggesting that infection with a live influenza virus may offer substantial cross-lineage protection in pigs against viruses of the same HA and/or NA subtype in pigs (40). Similarly, prior exposure to H1N1 or H3N2 conferred cross-protection against H1N2 in pigs in the absence of detectable hemagglutination inhibition and virus-neutralizing antibodies but inhibitory anti-neuraminidase (NA) antibodies were detected prior to infection with H1N2 (41). In contrast, in another study, pigs previously infected with H1N1 and subsequently challenged with H3N2 by aerosol did not develop fever, showed reduced virus shedding, and did not transmit to contact pigs (42). These conflicting results and the differences in heterotypic protection between the studies could be due in part to the different inoculation methods used, the dose and strain of the first and second exposures, the interval between the first and second exposure, and the degree of conservation of the internal genes between the strains used.

Taken together, these findings indicate that the mechanisms of heterotypic protection in mice and pigs may differ. This is supported by studies with another broadly protective influenza vaccine candidate, S-FLU, which induced heterotypic protection in mice and ferrets against a heterologous virus, while in the pigs, only lung pathology was reduced but not viral shedding (21, 26, 28). Cross-reactive immunity could be due not only to T cell responses to conserved internal antigens but also to antibodies to conserved epitopes of the haemagglutinin (HA) and neuraminidase (NA) (43).

Although we confirmed the potency of Ad-IL-1β as a mucosal adjuvant for enhanced local immunity, we observed higher gross- and histopathology scores. The samples for histopathology were taken from three different lung lobes (cranial, medial, and caudal right lung lobes) and although the histopathological lesions were similar in all groups, they were more extensive in the Ad-HA/NP+Ad-IL-1β-immunized pigs. We considered that the increased pathology may be due to vaccine-associated enhanced respiratory disease (VAERD) but the lack of increase in eosinophil numbers or larger perivascular cuffs in the lung did not support this.

VAERD was previously reported after the immunization of pigs with a whole inactivated H1N2 virus vaccine followed by infection with an antigenically mismatched pH1N1 virus. Non-neutralizing, vaccine-induced anti-HA2 antibodies promoting virus fusion were proposed as an underlying mechanism of the enhanced disease (44). More recently, Kimble et al. showed that mismatched immunization and challenge in ferrets also led to VAERD despite similar viral loads (45). As in pigs, antibodies from VAERD-affected ferrets were preferentially bound to the HA2 domain of pH1N1. Although we have not specifically tested the binding to the HA2 domain, serum from the Ad-HA/NP+Ad-IL-1β group did not show enhanced binding to either the H3N2 virus or to recombinant H3 protein compared to the other groups, which makes this a rather unlikely explanation. Furthermore, we were not able to detect an increase in pH1N1 or H3N2 infection of MDCK cells in the presence of Ad-HA/NP+Ad-IL-1β serum (data not shown). However, cross-reactive antibodies to recombinant H5 proteins were detectable in all treated animals.

Alternatively, as a high dose of IL-1β can cause pulmonary inflammation, emphysema, and airway remodeling in the adult murine lung (46, 47) or side effects like ruffled fur and reduced activity (11), it is possible that the increased pathology seen in our pigs was due to a different effect of IL-1β. However, we have not observed any side effects during the immunization phase and IL-1β has been used without ill effects in pigs and bovines before. Intranasal delivery of recombinant porcine respiratory reproductive syndrome (PRRS) virus expressing IL-1β in pigs enhanced the antibody response and prevented clinical signs in comparison to parental PRRSV (48). Co-administration of recombinant bovine IL-1β and a modified Bovine Herpes virus (BHV-1) vaccine enhanced both humoral and cellular responses against BHV-1 (49, 50). However, our data suggest that including IL-1β as an immunological adjuvant must be used with caution.

It is thought that harnessing local T cell immunity is essential for the development of an effective broadly protective influenza vaccine. In mice, vaccines or influenza viruses that induce T cell immunity are protective against heterologous virus challenges. In contrast, our results indicate that, in pigs, neither prior pH1N1 influenza exposure nor immunization with Ad-HA/NP with or without Ad-IL-1β confer heterotypic protection. Therefore, there are differences between models, additionally confounded by the different methods of measuring disease between rodents and large animals. Nevertheless, it is clear that, in pigs, a reduction of pathology is observed when a very powerful local T cell immune response is induced, for example by aerosol administration of the S-FLU vaccine, but not viral shedding, in contrast to ferrets (21, 24). These conflicting results indicate that we should be cautious in interpreting data obtained by a single model and certainly in extrapolating to humans. Direct evidence that heterotypic immunity in humans is mediated by T cells is lacking as most studies are based on the detection of T cells specific against conserved epitopes of internal antigens correlating with partial protection against new influenza strains. The mechanism(s) of human heterotypic protection, therefore, remains to be convincingly established. Whether pigs or small animals better represent the way in which humans respond to influenza viruses also remains to be determined.

## Data availability statement

The original contributions presented in the study are included in the article/**Supplementary Material**. Further inquiries can be directed to the corresponding author.

## Ethics statement

The animal study was reviewed and approved by The Pirbright Institute Animal and plant health Agency.

## Author contributions

ET, RW, and MT designed the study and obtained the funding. MT, AS, SiS, and CT generated and produced the vaccine. ET, AS, BP, VC, AMc, RW and EV coordinated the animal studies and processed samples; AS, BP, SV-H, SeS, AMc, EV, WG AM and VM acquired, analyzed and interpreted the data. FS performed the pathological analysis. ET wrote the manuscript. BP, AS, MT wrote, reviewed and edited the manuscript. All authors contributed to the article and approved the submitted version.

## Supplementary material

Supplementary Figure 1 Porcine B cells. **(A)** Gating strategy for porcine B-cell subsets. Lymphocytes were gated according to FSC-A/SSC-A properties and doublets and dead cells excluded (top panels). Lower panel: total B cells were identified by co-expression of CD79α and Pax5 (blue gate) and further sub-gated. Plasma cells/plasmablasts were identified by the co-expression of Blimp-1 and IRF4 (green gate). Blimp-1^-^IRF4^-^ B cells were gated (black gate) and further analyzed for Bcl-6 expression. CD79α^+^Pax5^+^Blimp-1^-^IRF4^-^Bcl-6^+^ cells were classified as GC B cells (red gate) and the remaining CD79α^+^Pax5^+^Blimp-1^-^IRF4^-^Bcl-6^-^ were considered as a mixture of naïve, activated, and memory B cells (purple gate). **(B)** Frequencies of total B cells, plasma cells, and GC B cells. Top left: CD79α^+^Pax5^+^ total B cells were quantified within total live lymphocytes across organs and treatment groups. Lower left and lower right: as above but CD79α^+^Pax5^+^Blimp-1^+^IRF4^+^ plasma cells and CD79α^+^Pax5^+^Blimp-1^-^IRF4^-^Bcl-6^+^ GC B cells, respectively, within total B cells. Each symbol represents data from an individual pig of the different treatment groups. Asterisks indicate significant differences between treatment groups within one location (*, p ≤ 0.05)

Supplementary Figure 2 Phenotype of porcine antigen-specific CD8β^+^ T cells in tissues. **(A)** Expression of CD45RA and CCR7 by CD8β^+^ T cells isolated from the indicated tissues of control Babraham pig. **(B)** CD45RA and CCR7 expression in DFE-specific CD8β^+^ T cells. Depicted are mean frequencies (± SEM) of naïve (CD45RA^+^ CCR7^+^), TCM (CD45RA^-^ CCR7^+^), TDE (CD45RA^+^ CCR7^-^), and TEM (CD45RA^-^ CCR7^-^) of five or three (control) pigs per group (n=3-5). Statistical significances were analyzed by two-way ANOVA followed by Tukey’s Multiple Comparison Test.

Supplementary Figure 3 Serum IgG responses against recombinant H1, H3, H5, and H7 at postmortem four days post H3N2 challenge at 1:80 dilution of serum. Each symbol represents one animal. Error bars represent mean ± SEM of pigs per group (n=5).
