# Supplementary figures and images for: Effect of mucosal adjuvant IL-1β on heterotypic immunity in a pig influenza model
Source: Front Immunol. 2023 Apr 20;14:1181716. doi: 10.3389/fimmu.2023.1181716 (PMC10159270; doi:10.3389/fimmu.2023.1181716)

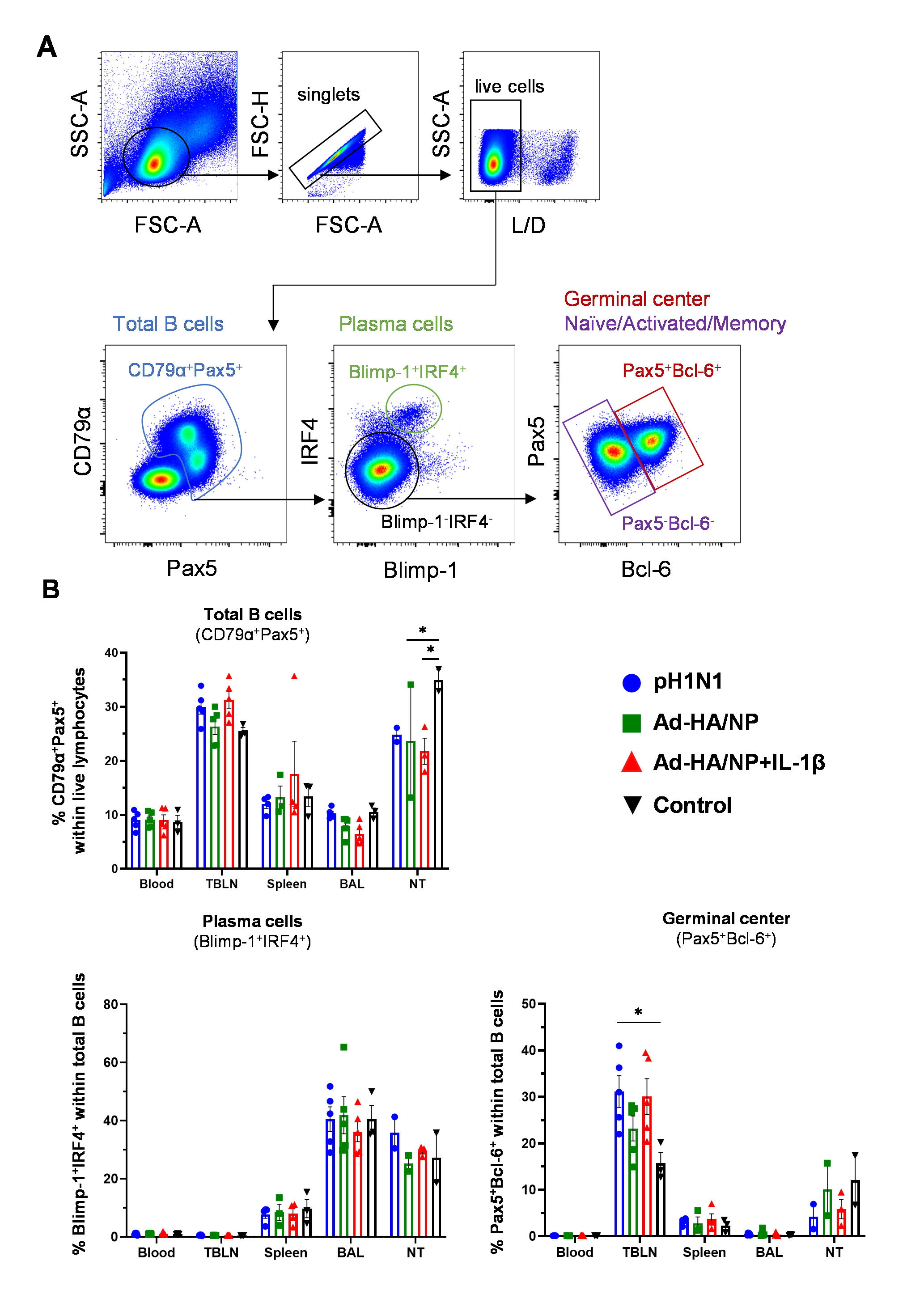

Supplement: Supplementary Figure 1 — Porcine B cells. (A) Gating strategy for porcine B-cell subsets. Lymphocytes were gated according to FSC-A/SSC-A properties and doublets and dead cells excluded (top panels). Lower panel: total B cells were identified by co-expression of CD79α and Pax5 (blue gate) and further sub-gated. Plasma cells/plasmablasts were identified by the co-expression of Blimp-1 and IRF4 (green gate). Blimp-1-IRF4- B cells were gated (black gate) and further analyzed for Bcl-6 expression. CD79α+Pax5+Blimp-1-IRF4-Bcl-6+ cells were classified as GC B cells (red gate) and the remaining CD79α+Pax5+Blimp-1-IRF4-Bcl-6- were considered as a mixture of naïve, activated, and memory B cells (purple gate). (B) Frequencies of total B cells, plasma cells, and GC B cells. Top left: CD79α+Pax5+ total B cells were quantified within total live lymphocytes across organs and treatment groups. Lower left and lower right: as above but CD79α+Pax5+Blimp-1+IRF4+ plasma cells and CD79α+Pax5+Blimp-1-IRF4-Bcl-6+ GC B cells, respectively, within total B cells. Each symbol represents data from an individual pig of the different treatment groups. Asterisks indicate significant differences between treatment groups within one location (*, p ≤ 0.05) [file Image_1.tif]

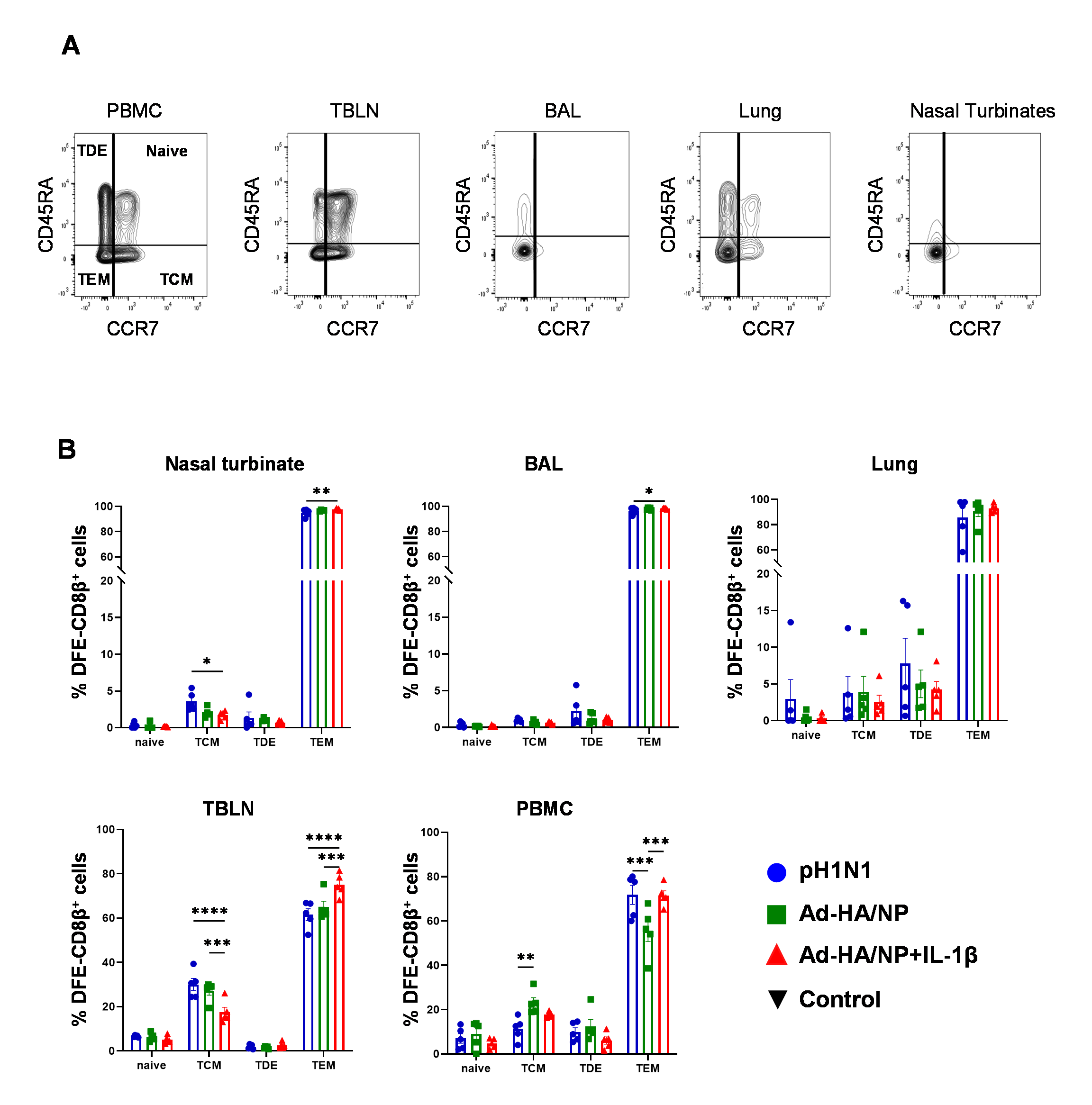

Supplement: Supplementary Figure 2 — Phenotype of porcine antigen-specific CD8β+ T cells in tissues. (A) Expression of CD45RA and CCR7 by CD8β+ T cells isolated from the indicated tissues of control Babraham pig. (B) CD45RA and CCR7 expression in DFE-specific CD8β+ T cells. Depicted are mean frequencies (± SEM) of naïve (CD45RA+ CCR7+), TCM (CD45RA- CCR7+), TDE (CD45RA+ CCR7-), and TEM (CD45RA- CCR7-) of five or three (control) pigs per group (n=3-5). Statistical significances were analyzed by two-way ANOVA followed by Tukey’s Multiple Comparison Test. [file Image_2.tif]

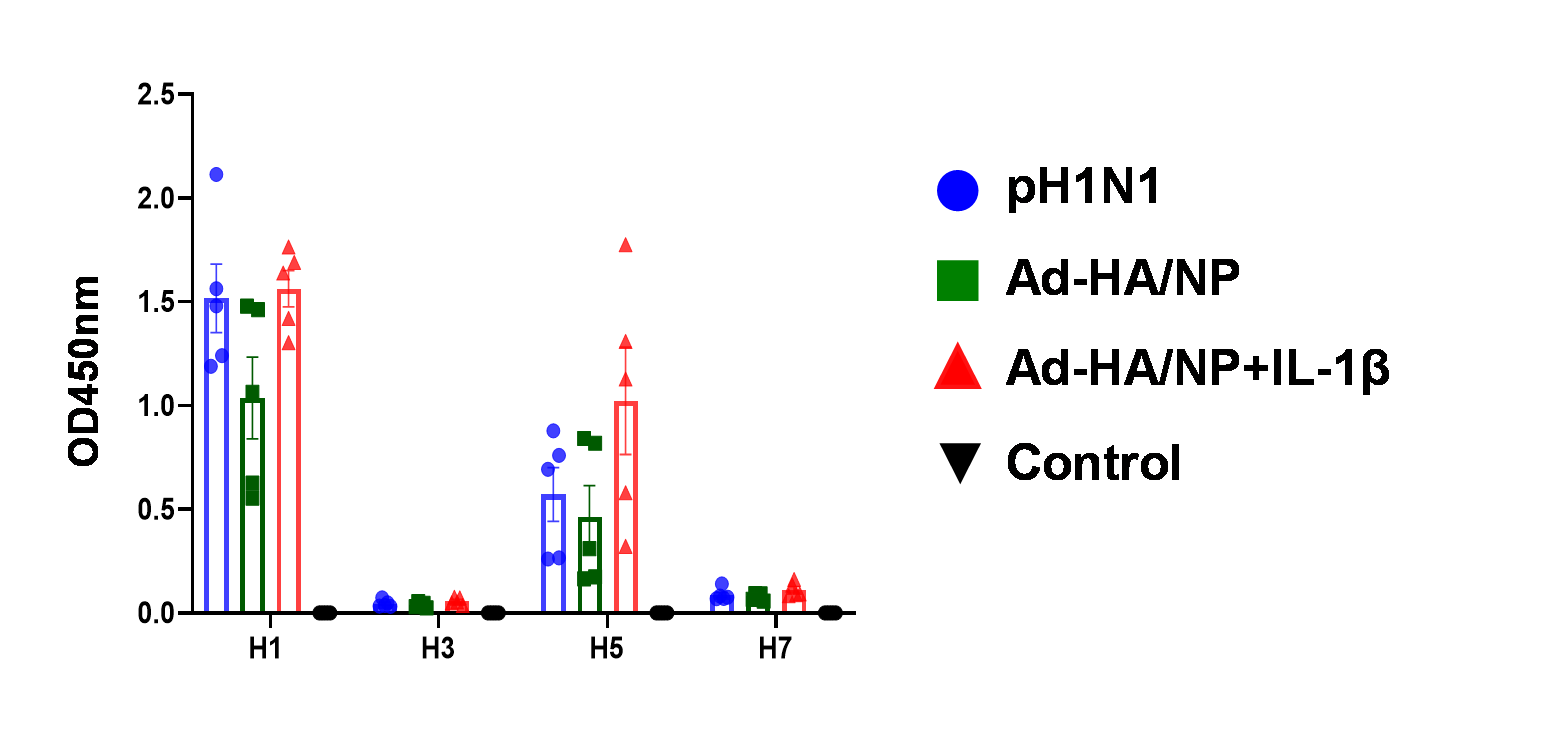

Supplement: Supplementary Figure 3 — Serum IgG responses against recombinant H1, H3, H5, and H7 at postmortem four days post H3N2 challenge at 1:80 dilution of serum. Each symbol represents one animal. Error bars represent mean ± SEM of pigs per group (n=5). [file Image_3.tif]
